# Post-tuberculosis lung disease: Towards prevention, diagnosis and care

**DOI:** 10.1016/S2213-2600(24)00429-6

**Published:** 2025-03-22

**Authors:** Jamilah Meghji, Sara C. Auld, Gregory P. Bisson, Celso Khosa, Refiloe Masekela, Neelima Navuluri, Andrea Rachow

**Affiliations:** 1 National Heart & Lung Institute, Imperial College London, London, UK; 2 Department of Respiratory Medicine, Imperial College Healthcare NHS Trust, London, UK; 3 Department of Medicine, Emory University School of Medicine and Departments of Epidemiology and Global Health, Emory University Rollins School of Public Health, Atlanta, GA, USA; 4 Department of Medicine, Division of Infectious Diseases, Perelman School of Medicine at the University of Pennsylvania, Philadelphia, PA, USA; 5 Instituto Nacional de Saúde, Marracuene, Mozambique; 6 Department of Physiological Science, Clinical Pharmacology, Faculty of Medicine, Eduardo Mondlane University, Maputo, Mozambique; 7 Department of Paediatrics and Child Health, College of Health Sciences, School of Clinical Medicine, University of KwaZulu Natal, Durban, South Africa; 8 Africa Health Research Institute, Durban, South Africa; 9 Division of Pulmonary and Critical Care, Department of Medicine, Duke University School of Medicine, Durham, NC, USA; 10 Duke Global Health Institute, Duke University, Durham, NC, USA; 11 Institute of Infectious Diseases and Tropical Medicine, LMU University Hospital, LMU Munich, Germany; 12 German Centre for Infection Research (DZIF), partner site Munich, Munich, Germany; 13 Unit of Global Health, Helmholtz Centre Munich, German Research Centre for Environmental Health (HMGU), Neuherberg, Germany

**Keywords:** Post-tuberculosis, Tuberculosis, Disability, Research Priorities

## Abstract

There is a growing body of data describing the high burden of respiratory sequelae experienced by TB survivors, including children, adolescents and adults. This group of sequelae, known as post-TB lung disease (PTLD), includes parenchymal damage, airway disease, and pulmonary vascular disease. It is thought that approximately half of pulmonary tuberculosis (PTB) survivors experience ongoing structural pathology, lung function impairment, or respiratory symptoms after the resolution of active disease. PTLD has been associated with adverse patient outcomes, including persistent symptoms and functional impairment, ongoing health seeking, and impacts on income and employment. There is much still to understand about the epidemiology and nature of PTLD, but in this manuscript we focus on strategies for prevention, diagnosis and care, to inform the ongoing work of TB-affected communities, healthcare providers, researchers and policymakers in this space. We summarise recent data, highlight evidence gaps, and suggest key research priorities for those working in the field.

## Introduction

TB disease remains a critical cause of morbidity and mortality worldwide, with an estimated 10.6 million incident cases in 2022.(1) Over 86% of those treated for TB disease survive,(1) but emerging data suggest a high burden of residual physical, psychological and socio-economic morbidity amongst TB survivors, even after treatment completion.(2, 3)

Factors contributing to this morbidity may include localised organ damage caused by TB-associated inflammation and fibrosis, the effects of systemic inflammation,(4) the side effects of anti-tuberculous medication,(2) and the broader socio-economic impacts of TB disease including stigma, social isolation, and loss of income and employment.(5, 6) Modelling studies suggest that almost half of the morbidity experienced in relation to TB disease globally may fall in the post-TB period.(7)

Although the importance of mitigating TB-associated catastrophic costs was captured in the End-TB strategy targets,(8) the need for TB programmes to measure and prevent broader TB-associated sequelae has not yet been recognised in the same way. This article focuses on post-TB lung disease (PTLD) as one important consequence of TB, which is relevant to the long-term well-being of pulmonary TB (PTB) survivors.(3)

## The burden of PTLD

**Figure 1:**
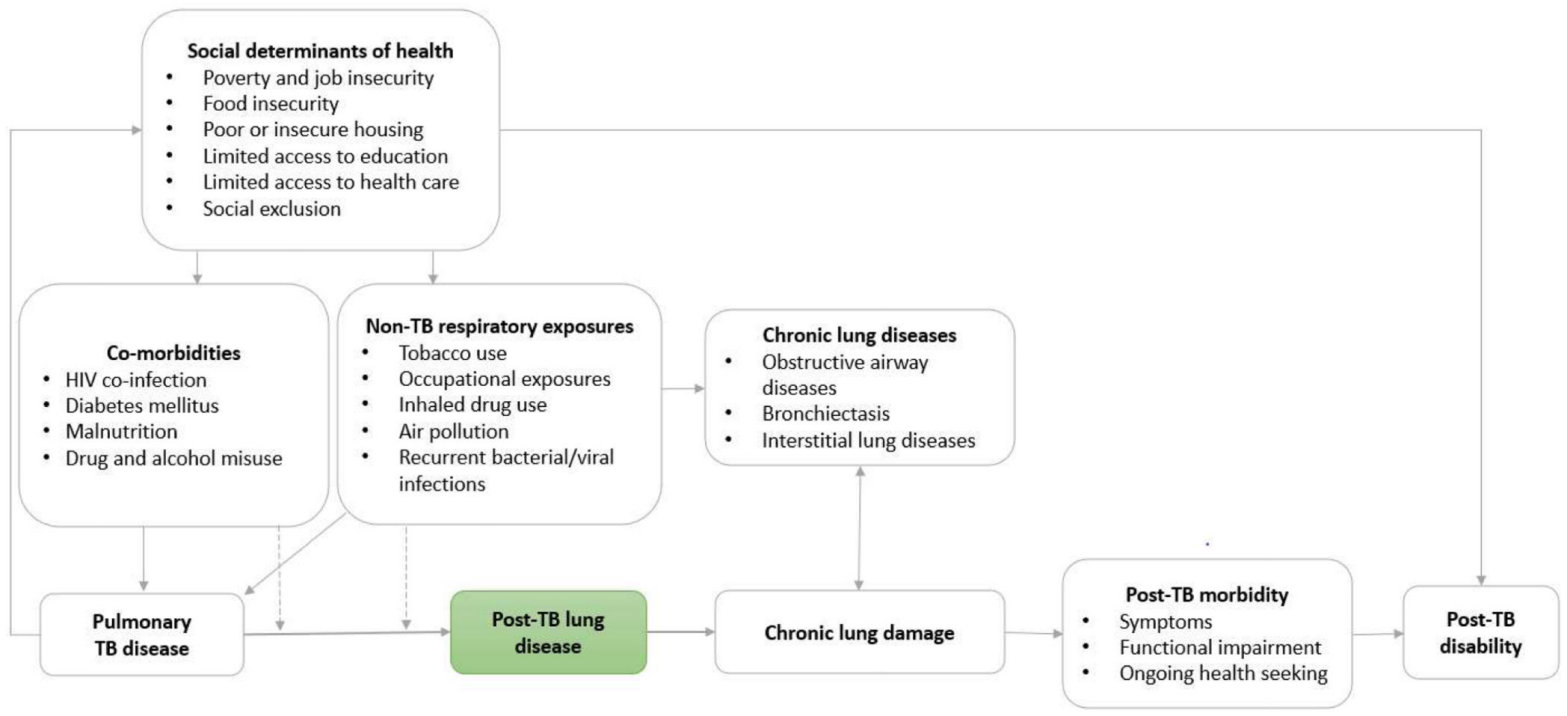
Conceptual framework describing potential drivers of PTLD, and associated impairment and disability Dashed lines denote potential effect-modification of the relationship between TB disease and the host immune response, and residual lung damage, by non-TB respiratory exposures and comorbidities.

The body of literature on post-TB lung disease (PTLD) has grown substantially since 2010, with the condition defined as “Evidence of chronic respiratory abnormality, with or without symptoms, attributable at least in part to previous tuberculosis”.(9) This is a pragmatic definition which recognises the broad range of respiratory damage caused by TB disease (Figure 1), and the heterogeneity of symptoms, impairment and disability resulting. It also recognises the challenge of differentiating lung damage caused by TB disease from that caused by other harmful respiratory exposures (E.g. smoking, occupational exposures, air pollution), which may be common in TB-affected communities.(10) However, there are no specific case definitions for PTLD for use in research, and studies have used diverse approaches to describe the prevalence of disease, behaviour over time, and secondary complications, making evidence synthesis challenging.

The majority of data on PTLD is drawn from low- and middle-income countries (LMICs), with relatively little data from high-income countries (HICs). The heterogenous patterns and severity of PTLD have been described in several narrative reviews.(3, 9, 11, 12) Three systematic reviews focused on lung function abnormalities after PTB disease suggest that approximately half of those treated for pulmonary TB (PTB) disease have abnormal spirometry at or after TB treatment completion,(13, 14) with severe disease in 10-15% of PTB survivors,(15) and a mixture of low forced vital capacity (FVC), obstructive, and mixed patterns of deficit observed.(14, 15) Those treated for multi-drug resistant (MDR) disease are more likely to have impaired spirometry and more severe disease than those treated for drug sensitive (DS) disease.(14, 15) Residual imaging abnormalities are common, with bronchiectasis observed in 35-86% of PTB survivors.(16, 17) Almost a quarter of TB survivors are symptomatic with a Medical Research Council (MRC) dyspnoea score of 3-5 at the end of treatment.(14) Few studies have described the relationship between HIV infection and residual PTLD, but some suggest that while HIV is associated with less severe PTLD,(16, 18) antiretroviral therapy (ART)-mediated immune reconstitution in those with TB-HIV co-infection may be followed by increased inflammation within the lungs, and loss of lung function.(19, 20) Work to date has not identified differences in the burden or nature of PTLD by sex. However, men experience a higher TB incidence than women, often present later with more advanced TB disease, and may experience gendered occupational exposures (E.g. Silica exposure through mining) which may further threaten their lung health. Investigation of the relationship between gender and PTLD may be highly relevant to strategies for prevention and care.

Prospective cohort data describing PTLD disease behaviour over time are limited, but show a trend to improvement in forced expiratory volume in the first second (FEV_1_) and FVC measures during TB treatment and in the 6-12 months after TB treatment completion, before plateau.(21) Up to a third of PTB survivors experience persistent pulmonary impairment or symptoms, with a possible sub-set of patients experiencing an accelerated lung function decline.(21) High rates of ongoing health seeking for respiratory complaints have been described in several settings, particularly in the first year after TB treatment completion.(16, 22) TB survivors face significantly increased mortality rates compared to the general population or TB-naïve controls even after TB treatment completion, with an estimated Standardised Mortality Ratio (SMR) of 2.9 (95% CI 2.2 – 3.8).(23–25). Where cause of death data are available, cardiovascular disease, cancer and respiratory diseases are amongst the leading causes of death for TB survivors. This is consistent with the increased burden of cardiovascular morbidity and lung cancer observed amongst PTB survivors compared to those who have not been treated for TB disease.(23, 26)

## Causal pathways underlying PTLD

The causal pathways underlying the heterogeneous prevalence and patterns of PTLD seen amongst TB survivors remain poorly understood. The pathogenesis of PTLD has been described in detail elsewhere, and likely relates to the burden and site of mycobacterial infection, the duration of infection and rate of clearance, the nature and force of the host immune response, and the healing response seen within the lung, including the potential for excessive remodelling and fibrosis.(27) The role of broader respiratory exposures including inhaled tobacco and drugs, occupational dusts and fumes, indoor and outdoor air pollution, bacterial or viral co-infection, and poverty-related factors such as nutrition in shaping the extent and pattern of lung damage during TB disease remains unclear. It is challenging to differentiate between the direct effect of these exposures on the lung, and damage mediated by TB disease, amongst TB survivors. Of note, it is not just the extent or pattern of lung damage sustained during TB disease, but the symptoms and functional impairment that result from this damage, as well as access to health services for ongoing care, and the broader physical and social context in which people live and work which shape experiences of post-TB disability in different settings (Figure 1).(5)

As is clear from this summary, the body of data describing the nature, drivers and impacts of PTLD has significantly grown in recent years. However, data on interventions to support the prevention, diagnosis, and management of PTLD at either the population or individual patient level remain scarce. In this review, we will highlight evidence gaps and research priorities in these critical areas with the hope of accelerating work in these spaces.

## Towards prevention

Interventions that minimise the lung damage sustained during PTB disease and treatment will be critical to preventing post-TB respiratory morbidity. Strategies may include public health interventions to ensure early TB diagnosis and treatment, as well as the clinical use of novel TB treatment approaches, or host-directed therapies. However, evidence in this space remains lacking, and sustained investment in clinical trials and intervention studies will be needed to generate robust data.

### Addressing the social determinants of TB disease

Upstream interventions which address the social determinants of TB (E.g. overcrowding, malnutrition) may be critical to mitigating PTLD, either by directly reducing the incidence of TB disease, or by modifying the extent or pattern of TB-related lung damage. The harmful impact of poverty-related risk factors on lung development are well described,(28) and those that experience these exposures will likely have less respiratory reserve to manage a ‘second hit’ of TB disease. However, data on the impact of non-pharmacological interventions to minimise PTLD remain limited. For example, whilst the RATIONS trial demonstrated the efficacy of macronutrient (food rations) and micronutrient support in reducing TB incidence and mortality amongst household contacts of individuals with microbiologically confirmed pulmonary TB, it did not report on respiratory outcomes.(29)

### Early TB diagnosis and treatment

Longer time to TB diagnosis is associated with more extensive lung damage or pathology on chest x-ray (CXR) at TB diagnosis,(30, 31) which in turn is associated with more extensive lung pathology at TB treatment completion.(32) It is therefore likely that measures to promote early diagnosis and treatment of TB disease will reduce the burden of PTLD at or after treatment completion.(33) The cost-effectiveness of interventions to promote early TB diagnosis would be further strengthened if they could be shown to minimise post-TB sequelae, as well as improving TB case detection rates.

Active case finding (ACF), in which persons who are not seeking health care for symptoms are invited to be screened for TB disease, holds much promise. A trial of South African gold miners randomized to either 6- or 12-monthly radiological screening showed no difference in the TB detection rates, but those screened more frequently had significantly less extensive disease on chest radiograph.(34) Although this is a specific sub-population with concurrent silica exposure, this suggests some possible benefit of active screening to enable early diagnosis and mitigate lung damage. However, broader ACF interventions have largely been evaluated from a population health perspective by measuring change in community TB prevalence over time,(35) and none of the recent trials of community-based ACF programs compared the extent of CXR pathology or other respiratory measures at or after TB treatment completion between active and passive case finding arms.(36, 37)

In recent years there has also been growing recognition of subclinical TB disease, in which individuals are asymptomatic but have bacteriologically confirmed TB, as a substantial contributor to TB transmission.(38) It remains unclear how many individuals with subclinical disease will go on to develop symptomatic, clinical TB disease.(39) However several studies indicate that those with subclinical disease have relatively minimal radiographic involvement: a recent individual-participant data meta-analysis reported that only 2.8% (970/35,241) of those without any cough had positive findings on chest radiograph,(40) whilst a population-based cohort study from Canada found absent or minimal radiographic findings in 86.4% (247/286) of patients with subclinical disease.(41) Diagnosis and treatment of patients at this early stage of disease may prevent progression of damage and the development of lasting PTLD.

### The potential role of novel TB treatment regimens

The pursuit of novel antimicrobial drug regimens which are shorter, safer and simpler is ongoing, with a move towards individualised TB regimens tailored to patients’ comorbidities, and the site and extent of TB disease.(42) However, we are not aware of any modern randomized clinical trials of antitubercular therapy that have prioritised the reporting of pulmonary impairment after treatment as a primary outcome.

Trials of new TB treatment strategies often use sputum culture conversion, for example at 2 and 6 months, and time to culture conversion as markers of disease activity or predictors of relapse-free cure.(43–45) However, given that pulmonary damage in the context of TB disease is likely primarily driven by host-mediated inflammation, rather than directly by mycobacterial factors,(27) it remains uncertain whether improved microbiologic metrics such as early bactericidal activity and faster culture conversion will necessarily be correlated with the pattern or severity of PTLD. The recent TRUNCATE TB trial, an adaptive platform trial of shortened courses of treatment for drug-susceptible TB, included chest radiographs, spirometry, and assessment of respiratory symptoms at 96 weeks as secondary outcomes and did not find substantial differences between treatment arms.(46) Another area of interest for reducing long-term pulmonary morbidity is the use of inhaled antimicrobials, which may have better activity in areas with extensive tissue destruction and cavities where drug penetration from the blood compartment may be particularly poor. A small clinical trial from Thailand found that participants randomized to receive an inhaled dry powder formulation of isoniazid, rifampicin, pyrazinamide, and levofloxacin in addition to standard TB treatment had no significant difference in sputum culture conversion at 8 weeks but did have more rapid resolution of cough and a trend towards more rapid improvement in the extent of disease on chest radiograph.(47) Additional trials will be needed to confirm these preliminary findings and their implications for PTLD.

### Modifying the host immune response

Host-directed therapies (HDTs) are adjuvant treatments given alongside antimicrobials to modify the host immune response. Broadly, these therapies aim to reduce pathology and/or improve bacillary killing. Particular agents of interest include those that modify eicosanoid pathways (E.g. Non-steroidal anti-inflammatory drugs (NSAIDs), lipoxygenase inhibitors), reduce inflammation (E.g. Corticosteroids, ibuprofen, N-acetylcysteine (NAC)), improve autophagy and intracellular bacillary processing (E.g. Metformin, statins, tyrosine kinase inhibitors), antifibrotics, and agents that modulate the extracellular matrix and associated proteases, including matrix metalloproteinases (MMPs).(27) HDTs for TB have been the subject of recent reviews,(48, 49) but are discussed below in the context of respiratory health outcomes.

Corticosteroids have been widely evaluated, with many studies completed prior to the use of rifamycin-based therapies. A systematic review and meta-analysis of randomized trials from 1959 to 1999 found more rapid radiographic resolution of pulmonary infiltrates and higher rates of cavity closure in those treated with steroids, particularly during the initial months of treatment.(50) However an updated analysis including studies from 1966 to 2014 identified a positive effect of steroids in only two of five trials reporting lung function outcomes over the TB treatment period.(51) A sub-analysis of the Pred-ART Trial, which randomized HIV-infected, ART-naïve adults with CD4 cell counts ≤100 cells/μl diagnosed with PTB to four weeks of prednisone or placebo alongside antimicrobials, showed more rapid improvement in spirometry, six-minute walk test, and symptom-related quality of life between baseline and week four in the intervention group, but these differences were not observed after steroids ceased.(52) Taken together, steroids may improve clinical manifestations of pulmonary inflammation either early in treatment or during administration, but consistent evidence of sustained benefit are lacking.

Several trials of broader HDT agents are ongoing. A randomized clinical trial of the type 4 phosphodiesterase inhibitor CC-11050, an anti-inflammatory agent, in HIV-uninfected adults with moderate to severe radiographic involvement at PTB diagnosis showed greater recovery of FEV_1_ at day 180 compared to controls.(53) A similar effect was seen with everolimus, an inhibitor of the protein kinase mTOR which may reduce inflammation and fibrosis, within the same trial.(53) Effects attributed to these drugs were approximately twice the magnitude (~6%, or 200mL) considered clinically meaningful in COPD trials and, in contrast to the effect observed in trials of corticosteroids, appeared only later during follow-up. Statins have been associated with accelerated bacterial clearance in preclinical models,(54, 55) and reduced rates of incident TB disease in clinical studies,(56) but their effect on PTLD remain unclear. A phase 2b randomized trial of rosuvastatin given for 8 weeks during TB treatment showed no difference in the change in FEV_1_/FVC ratio, chest radiograph changes, or quality of life scores from baseline to week 24 between intervention and control arms.(57) In contrast, a randomized trial of atorvastatin demonstrated a greater reduction in CXR severity score in the statin versus standard of care arms.(58) More rapid resolution of imaging and cavity closure has also been observed in a small trial of the MMP inhibitor doxycycline(59), and adjunctive N-acetyl cysteine (NAC) has been associated with improved lung function recovery.(60) Trials evaluating ibuprofen and acetylsalicylic acid as anti-inflammatory HDTs for TB are ongoing (NCT04575519).

## Towards diagnosis

There is little consensus about when and how TB patients and survivors should be evaluated for residual respiratory morbidity. Routine surveillance of PTLD within TB programmes would provide data on the local burden of disease for research purposes and would inform health service planning for post-TB care, whilst at the individual level, the diagnosis of PTLD would support linkage to care. However, it has been challenging to agree on standardised approaches to PTLD measurement within TB services given its heterogeneity, the lack of prospective data on the patterns of PTLD associated with adverse clinical outcomes, and limited data on the implementation of screening and diagnostic approaches in real-life settings.

### Heterogeneity of PTLD

TB disease may affect multiple pulmonary compartments, including the lung parenchyma, large and small airways, vasculature and pleura (Table 1). Severe disease may also cause deconditioning and respiratory muscle weakness. Diverse patterns of pathology may be observed between people with PTB disease, but also within the lung tissue of a single individual,(16) making measurement of PTLD as a single clinical entity challenging. Symptoms also vary widely – some individuals with abnormal imaging or lung function may be asymptomatic, whilst others may experience shortness of breath, cough, chest discomfort, or sputum production. The limited and variable correlation between lung function, imaging, and respiratory symptoms observed within individuals with PTLD makes the accurate identification of PTLD with a single diagnostic tool challenging.(61) Lastly, patterns and severity of PTLD evolve over time, even after treatment completion, with diverse trajectories observed between individuals, thus raising questions about the timing of diagnosis.(21, 62, 63) The majority of individuals experience some recovery in lung function and imaging in the year after TB treatment completion, likely driven by resolving inflammation and tissue remodelling.(21) However, FDG PET-CT studies demonstrate persistent or new metabolic activity within the lung during this year, suggesting a possible role for ongoing tissue inflammation, and there may be a number of TB survivors who experience accelerated lung function decline over this period.(21, 64) These three issues – the heterogeneity of patterns of PTLD, the absence of a single approach to measuring disease, and diverse and ongoing evolution over time – make decisions about when to investigate for PTLD and what tools to use challenging for both clinicians and researchers.

Of note, there is growing interest in the concept of TB-endotypes – distinct patterns of immunological and molecular mechanisms which vary between people, and drive TB disease heterogeneity. As our understanding of the links between TB pathogenesis, TB treatment response, and lung recovery grow, it may be that this concept could be extended to the clinical manifestations of PTLD, such that we are able to develop more nuanced diagnostic biomarkers or categories of PTLD which will facilitate a more precision-medicine based approach to diagnosis and care.

### Implementation challenges

Despite these challenges, several countries are now considering including PTLD surveillance or individual-level screening within national TB guidelines.(67, 68) These screening approaches must be low-cost, acceptable, reliable, and feasible to implement if they are to be used by decentralised TB services in low-resource settings, and there may be efficiency in using existing TB diagnostic tools (E.g. chest radiographs) to support this. There may be benefits from implementing screening for lung damage during TB treatment, to support linkage to early intervention to mitigate respiratory damage and improve recovery. Screening at existing TB clinic visits may also be more efficient from both a patient and provider perspective. Ideally PTLD screening should capture individuals at greatest risk of adverse outcomes. However, longitudinal data on PTLD outcomes remain limited and it has proven challenging to identify the predictors of adverse post-TB outcomes within existing datasets. The pros, cons, and data gaps use for various tools which could be used for surveillance and screening are shown in Table 2.

More advanced respiratory diagnostics such as chest computed tomography, body plethysmography or gas transfer may better differentiate between PTLD-specific pathological patterns or phenotypes.(16, 62) However, whilst the inclusion of these tests in clinical research studies will advance our understanding of pulmonary pathology and the long-term cardio-respiratory sequelae of PTLD, they are unlikely to be widely available in the majority of high TB burden settings in the near future.

## Towards care

As evidence on the burden and impact of PTLD emerge, there are growing calls for interventions to improve post-TB care. Both Clinical Standards and Expert statements have been produced to guide this process,(9, 69, 70) but primary data on the clinical impact of interventions to manage established PTLD, prevent and manage secondary complications, and address the high burden of recurrent TB disease seen amongst TB survivors remain scarce. Given the heterogeneity of PTLD disease, it may be that an approach addressing ‘treatable traits’ for individual patients will be required. The potential benefit of routine follow-up of TB survivors after TB treatment completion in the absence of robust interventions remains unclear and may incur health system and patient costs. Data on the preferences of TB survivors regarding follow-up timing, duration, and approach are urgently needed to inform these approaches.

### Managing established PTLD

There are no evidence-based guidelines for the management of those with established PTLD, whether they are diagnosed at TB treatment completion, or they return with symptoms or complications months or years later. Interventions proposed to date include nutritional support (micronutrients or calorie supplementation) to promote recovery, chest physiotherapy and pulmonary rehabilitation, smoking cessation, inhaled therapies, oxygen therapy and respiratory support, and surgical resection of destroyed lung tissue. Most of these suggestions are rooted in the morbidity and mortality benefits seen in other chronic respiratory diseases, but there remains little robust data on their clinical impact, feasibility and cost-effectiveness in PTLD specifically.(71, 72)

Pulmonary rehabilitation (PR) is a low-cost intervention with growing interest and evidence in populations with PTLD. It can be delivered in community settings, has been shown to have a mortality benefit in other chronic lung conditions, may address the general deconditioning and muscle loss experienced during TB disease, and has been included in published Clinical Standards for PTLD care.(69) However, even here data on long-term impact are scarce.(71, 72) A 2023 review identified seven studies of PR reporting on patient outcomes after TB treatment completion, including only two small trials (n=60 and 62), and evidence on the sustained effect over time were limited.(71)

While inhaled steroids and bronchodilators are often used for PTLD, the pathophysiology of phenomena such as airway obstruction may be different in those with TB versus smoking-related disease, and it will be important to obtain direct evidence of the role of these treatments amongst TB survivors, rather than extrapolating findings from broader COPD studies. This is particularly relevant given the high cost and stigma associated with inhaled therapies in many low-resource, high TB burden settings,(73) and highlights the need to include person-centred outcomes (E.g. Quality of life measures) or qualitative data when designing and evaluating interventions for post-TB care.

### Managing secondary complications

Secondary complications of PTLD may include secondary pulmonary infections, pulmonary hypertension and cor-pulmonale,(66) lung cancer, (74) and in severe cases respiratory failure.(75) Data on the incidence and time to these complications remain limited, but they are well described in case series and well recognised by clinicians in high TB incidence settings. Post-TB cardiovascular disease is increasingly recognised and was the leading cause of death in a recent meta-analysis of post-TB mortality.(23)

TB survivors with bronchiectasis or destroyed parenchyma may be at particular risk of secondary pulmonary infections. There are limited data on the microbiology of bronchiectasis amongst TB survivors. Studies of patients with all-cause non-CF bronchiectasis in high TB burden settings such as India where over a third of disease is likely TB-related report a high burden of gram-negative infections,(76) but significant geographical variation is likely. PTB survivors with residual cavities or destroyed lung are likely at increased risk of fungal lung disease, including aspergilloma and invasive pulmonary aspergillosis. Whilst existing data suggest moderate rates of Aspergillus IgG seropositivity amongst TB survivors,(77, 78) data on the prevalence, patterns, and time to clinically relevant fungal lung disease remain limited. The incidence of non-tuberculous mycobacterial (NTM) pulmonary disease in high TB incidence settings is poorly described in general, including amongst TB survivors.

Further data will be needed to inform our approaches to the prevention, diagnosis and management of secondary infections amongst TB survivors. Vaccination against respiratory pathogens including influenza, COVID-19, and pneumococcus have been suggested at TB treatment completion,(3) rooted in our understanding of the importance of this for the prevention of disease in broader populations with chronic respiratory diseases, but data on infection microbiology, infection risk, and vaccine immunogenicity will be needed to determine the clinical efficacy and potential timing of respiratory vaccinations in this patient group.

### Prevention and diagnosis of recurrent TB disease

TB survivors are at risk of recurrent TB disease through either endogenous relapse or exogenous reinfection,(79) and surveillance data from several African sites have shown that previous TB disease remains a dominant risk factor for a new TB diagnosis.(80, 81) A systematic review and meta-analysis of 175 studies found a pooled estimate of TB incidence of 2.26 per 100 person years at risk (95% CI 1.87-2.73) amongst TB survivors, with a mean follow-up of 2.3 years.(82) However, rates of recurrence vary widely between settings, with higher rates in high TB-incidence settings, and amongst those treated for drug-resistant TB or living with HIV.(83) Disease relapse remains the most common cause of recurrent TB disease, accounting for 70% (95% CI 42-74%) of cases within a review of 48 studies that used DNA fingerprinting, but again with marked heterogeneity between settings.(82)

The relationship between post-TB structural lung damage, TB relapse, and TB reinfection remains unclear. Cavitary TB disease has been associated with higher rates of treatment failure and disease relapse.(84) This may reflect a high burden of bacterial disease in those with cavitation, and issues around drug penetrance during the initial TB treatment episode, rather than the effect of structural lung pathology on post-TB immunity. Recurrent disease amongst TB survivors may also reflect high rates of re-exposure amongst those returning to similar social environments or unchanged underlying social determinants of health (E.g poverty or poor access to care). Work exploring the effect of TB disease and treatment on patterns of systemic inflammation, the host immune response, and the microbiome, and the impact this may have on post-TB infection is ongoing.(85)

The diagnosis of recurrent disease may be challenging amongst PTB survivors, many of whom experience residual or recurrent respiratory symptoms due to PTLD or secondary bacterial or fungal infections. Empirical antibiotics are often used as part of the diagnostic pathway for recurrent TB disease, but there is little microbiology data to guide the choice of antibiotic for PTLD exacerbations, and a lack of data to support the use of empirical antibiotics as part of the TB diagnostic pathway in general.(86) The challenge of diagnosing recurrent TB disease is exacerbated by the reduced specificity of nucleic acid amplification tests such as Xpert MTB/Rif – increasingly used as a primary TB investigation instead of sputum smear – amongst TB survivors compared to TB-naïve adults, particularly within 2 years of treatment completion.(87, 88) The specificity of Xpert Ultra is likely lower than that of Xpert MTB/Rif in this patient group.(89) First line use of CXR for the diagnosis of recurrent TB disease may be similarly challenging in the context of residual structural pathology after a first episode of TB disease, and artificial intelligence (AI) algorithms for TB diagnosis on CXR frequently do not account for previous TB disease.

## PTLD amongst children and adolescents

Children represent approximately 11% of the global TB burden.(1) The patterns, severity, presentation and outcomes associated with PTLD in children and adolescents depend on the timing of the lung insult, the developmental stage and maturity of the immune system, and the potential for lung recovery with lung function ‘catch-up’ over time.(90) Lung damage during childhood may track over the life course,(90) and PTLD amongst young children may be a significant contributor to the overall years lived with disability (YLDs) and burden of TB disease.(7) TB disease during childhood and adolescence may also have critical and lasting effects on educational, social, psychological well-being.(91)

Children have different presentations of TB disease compared to adults, with a higher burden of pauci-bacillary disease and primary complex TB, and the nature of the residual lung damage may differ accordingly. Patterns of pathology seen amongst children and adolescents include bronchiectasis, and obstructive and restrictive lung disease, with symptoms including cough, wheeze, shortness of breath, chest pain, increased work of breathing and stridor.(92) Birth cohort data from South Africa – although potentially confounded by socio-economic status – suggest that young children treated for TB disease have a higher prevalence of wheezing and lung function deficits by age 5-years compared to TB-naïve children, with greater impairment amongst those diagnosed before 1-year of age.(93) Cross-sectional data from The Gambia show lower lung function indices, higher prevalence of respiratory symptoms, and lower quality of life scores amongst older children previously treated for TB disease compared to age-matched household controls.(91) A prospective study of adolescents in South Africa showed lower spirometry indices and more gas trapping amongst those completing TB treatment compared to household controls.(94) A recent systematic review and meta-analysis identified 5 studies including data from 567 children ≤18 years at varying times from TB treatment, and suggest sustained deficits in FEV_1_ z-scores (−1.53 (95% CI −2.65, –0.41)) and FVC z-scores (−1.93 (95% CI −3.35, –0.50)), but with significant heterogeneity.(95)

As with adults, there is a lack of longitudinal data on change over time, limited data on the burden and time to secondary complications, and little consensus about strategies for surveillance or screening for PTLD amongst children. The diagnosis of PTLD is particularly challenging amongst children – lung function assessment can be difficult in young children, and spirometry has limited sensitivity to detect early damage. Approaches such as tidal breathing maneuvers, impulse oscillometry, and multiple breath washout may be more easily completed and sensitive but are not widely available in high TB burden settings. Access to CXR imaging may also limited for many children. Observational work to increase our understanding of PTLD amongst children and adolescents is ongoing, and interventional studies to develop feasible approaches to screening and management are much needed.

## Health systems perspectives

**Figure 2:**
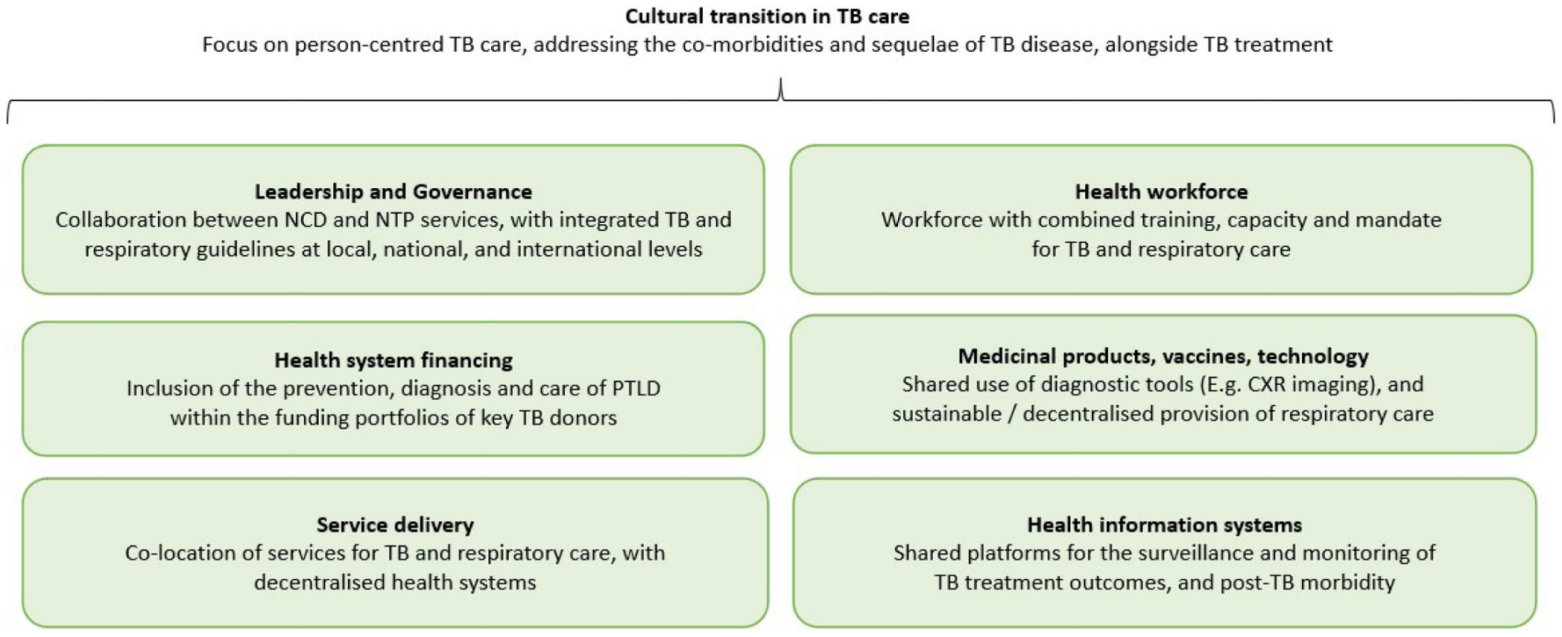
Changes required to the Health System pillars, to delivery PTLD prevention, diagnosis and care PTLD – Post-TB lung disease; NCD – Non-communicable disease; NTP – National TB Programme; CXR – Chest radiograph

Relatively little is known about the health systems which will be needed to deliver prevention, diagnosis and care for PTLD. The identification of residual morbidity at TB treatment completion may fall within the remit of National TB Programmes (NTPs), but long-term respiratory care will likely require collaboration with non-communicable disease (NCD) services.(70) Basic PTLD services may need to be decentralised and delivered at the point of TB care, and gender-specific challenges around access to care will need to be taken into account if we are to ensure service delivery. Post-TB services may be more sustainable if delivered using an integrated approach, which includes both TB and respiratory care, rather than as an additional vertically implemented service.(96) However, this will require a shift in both the culture and delivery of TB services (Figure 2).

The paucity of existing models of joint TB-NCD or TB-respiratory care has previously been a key barrier to implementation of integrated services.(97) Whilst the approaches required to achieve this will likely be context dependent,(98) there may be opportunities to learn from to the example of integrated HIV-NCD care in LMICs.(99) Several countries have included operational research around post-TB morbidity screening and care in funding applications to The Global Fund as part of the 2023-2025 funding cycles, whilst others have incorporated post-TB services within national guidelines for TB patient care,(67) and more robust data on implementation may be available in the near future. Improved community awareness and literacy about PTLD is likely to be critical to the implementation of these services, particularly given the stigma associated with TB disease,(100) respiratory symptoms such as cough,(101) and respiratory treatments such as inhalers in many settings.(73)

## Conclusion

There is now a convincing body of data describing the prevalence and patterns of residual lung damage amongst TB survivors, and the marked impact this may have on the lives and livelihoods of TB-affected households. However, critical gaps remain in our understanding of pathogenesis and prevention, approaches to screening and diagnosis, and data on the impact, cost and feasibility of strategies for post-TB care. This is particularly important for children and adolescents, in whom respiratory damage may persist over the life course. Further observational data are needed to describe causal pathways and disease behaviour over time, interventional work will be needed to address gaps in prevention and care, and a health systems focus will be needed to support feasible and sustainable implementation across all age groups (Table 3).

Although this review has focused on PTLD, this is only one example of post-TB morbidity. Further work will be needed to describe the sequelae of extra-pulmonary disease including TB meningitis or musculoskeletal disease, and the psycho-social and economic morbidity experienced by many TB survivors, as well as to understand the broader social contexts which shape peoples’ experience of post-TB disability. Research and implementation work will be needed across these areas, if we are to truly improve the long-term wellbeing of TB-affected communities.

## Figures and Tables

**Table 1:** Observed patterns of TB-related lung damage

	Primary pathology
Parenchyma	Cavitation Parenchymal destruction Emphysematous change Atelectasis Fibrosis with volume loss / anatomical distortion
Airways	Bronchiectasis Small airways disease Obstructive airway disease
Pleura	Pleural thickening and calcification
Pulmonary vasculature	Venous thromboembolism(65) Pulmonary hypertension(66)

**Table 2:** Potential tools for PTLD surveillance/screening

Tool	Pros	Cons	Priority data needed
Symptom screening	Low cost Quick Does not require specialist training to administer Potentially strong predictor of patient outcomes Could be used alongside questions for broader morbidity (e.g. anxiety and depression) Identifies symptoms, which are a patient-focused outcome	Limited sensitivity – may miss asymptomatic PTLD Limited specificity – may capture non-respiratory causes of dyspnoea and may not differentiate between PTLD phenotypes. Does not differentiate between patterns of disease - limited information to inform treatment decisions Subject to recall bias	Relationship between end of treatment symptoms and long-term morbidity Validity and inter/intra observer variability of different symptom questions or tools Sensitivity/specificity of a PTLD-specific symptom screening tool compared to existing tests, for specific PTLD phenotypes
Exercise testing	Low cost Requires limited staff training Identifies functional impairment, which is a patient-focused outcome	Time consuming Requires space and a standardised testing environment Interpretation dependant on reference ranges Limited sensitivity – may detect only severe disease Limited specificity – may capture non-respiratory causes of dyspnoea, and may not differentiate between PTLD phenotypes Does not differentiate between patterns of disease - limited information to inform treatment decisions Limited to patients that can walk/stand	Relationship between end of treatment functional capacity and long-term morbidity Validity of different exercise tests in different PTLD phenotypes Reference values for populations in high TB-burden settings
Spirometry	Sensitive Non-invasive Relatively specific to respiratory pathology Allows differentiation of patterns of disease, and may inform treatment decisions Associated with adverse health outcomes in broader respiratory conditions (E.g. COPD)	Time consuming Requires dedicated trained staff Requires specialist equipment Highly operator dependant and requires quality control and oversight Interpretation dependant on reference ranges	Relationship between patterns of deficit on end of treatment spirometry, and long-term morbidity Real-life feasibility data, describing the ability to perform and interpret spirometry in decentralised services outside of the research setting Reference values for populations in high TB-burden settings
CXR imaging	Sensitive Non-invasive Specific to lung pathology Equipment already in use for TB disease diagnostics Potential for interpretation by AI End of treatment imaging could be used as a baseline for investigations of recurrent TB disease Allows differentiation of patterns of disease, and may inform treatment decisions	Higher cost than alternatives CXR not yet available in all settings – may require additional equipment or investigation at another site Limited sensitivity - may not detect airways disease Requires trained staff or AI for interpretation	Relationship between patterns of pathology on end of treatment CXR, and long-term morbidity Development of (AI) algorithms for the interpretation of EOT (instead of diagnostic) CXR imaging
Oxygen saturation	Low cost Quick Equipment required is more broadly relevant for clinical care Relatively specific to respiratory pathology Focused on those with severe disease	Limited sensitivity – will miss mild-moderate disease Does not differentiate between patterns of pathology - limited information to inform treatment decisions	Relationship between hypoxia at end of treatment, and long-term morbidity

AI: artificial intelligence; CXR: chest x-ray; COPD: chronic obstructive pulmonary disease.

**Table 3:** PTLD research priorities, relevant to children, adolescents and adults.

Theme	Priority research areas
Towards prevention	- Studies exploring the causal pathways for PTLD, including effect modifiers of the relationship between TB disease and residual lung damage. - Data describing the impact of early TB diagnosis and treatment (including through TB active case finding interventions) on the incidence, severity and pattern of PTLD. - Data describing the impact of novel TB-treatment regimens and time to microbiological cure on the incidence, severity and pattern of PTLD. - Robust host-directed therapy trials which include respiratory outcomes at or after TB treatment completion.
Towards diagnosis	- Longitudinal data on post-TB respiratory outcomes, to determine trajectories over time, and to identify patient characteristics and disease patterns which can be identified early and are associated with adverse long-term outcomes. - Development of TB endotypes, linking pathogenic mechanisms, the patterns/severity of lung damage, and long-term patient outcomes. - Operational research data, describing the cost, feasibility, sensitivity, specificity and predictive values of different approaches to PTLD screening. - Development and validation of dedicated PTLD symptom screening and quality of life scores. - Development of AI algorithms for the diagnosis of PTLD from end-of-treatment chest radiographs.
Towards care	- Qualitative data describing patient perspectives on the use, duration, and approach to post-TB follow up and care, with a gendered perspective where appropriate. - Robust data on the risk, timing, and outcomes of secondary infections with bacteria, viruses, aspergillus, and NTM, particularly in low- and middle-income countries. - Data on the immunogenicity and clinical effectiveness of respiratory vaccines amongst TB survivors at TB treatment completion. - Diagnostic accuracy studies for recurrent TB disease amongst TB survivors, to inform the need for dedicated guidelines for TB diagnosis in this group. - Development of AI algorithms for the diagnosis of recurrent TB disease amongst TB survivors, using chest radiographs. - Data on the long-term clinical impact, feasibility, timing, and cost-effectiveness of key interventions to mitigate post-TB morbidity (E.g. Nutritional support, chest physiotherapy and pulmonary rehabilitation, and inhaled therapies)
Health systems	- Stakeholder perspectives on the financing, governance, service delivery, and monitoring of approaches for the diagnosis and management of PTLD. - Pilot studies of integrated TB and respiratory care for PTLD, with robust process evaluation of how these interventions work, for whom, and the impact on broader TB and NCD care.

## Search strategy and selection criteria

References for this narrative review were identified by the authors, based on data included in recently published peer-reviewed systematic reviews on PTLD, recent clinical statements/guidelines for post-TB care, evidence presented at the First and Second International Post-Tuberculosis (TB) Symposia, and their experience in this field. There were no restrictions on date or language of publication. No formal literature searches were completed to inform this review.

## Key messages

- People affected by TB disease face a high burden of physical, psychosocial, and economic sequelae after TB treatment completion.
- Post-TB lung disease (PTLD) is a heterogenous condition that includes residual damage to the airways and parenchyma from pulmonary TB disease and is associated with ongoing respiratory symptoms, functional impairment, and health-seeking after treatment completion.
- The burden of PTLD is increasingly well described in low-resource, high-TB incidence settings, but data on strategies for the prevention, diagnosis, and care of those with PTLD remain limited.
- Approaches to prevention may include measures to address the upstream social determinants of TB disease, support early TB diagnosis and treatment, novel TB treatment regimens, and host-directed therapies.
- Efforts to improve the accurate diagnosis of PTLD must address the heterogeneity of disease, and challenges around the decentralised implementation of respiratory diagnostics in low-resource settings.
- Post-TB clinical care must include the active management of PTLD itself, as well as the diagnosis and prevention of secondary complications (E.g. superimposed respiratory infections) and recurrent TB disease.
- Across all interventions for the prevention, diagnosis, and management of PTLD, it will be important to address the needs of children and adolescents, and to develop feasible, equitable and sustainable health systems for implementation.
